# An accurate semi-empirical model for PMT pulse signal analysis

**DOI:** 10.1371/journal.pone.0313999

**Published:** 2024-11-26

**Authors:** Shuo Li, Xudong Lyu, Fei Wang, Chao Sun

**Affiliations:** 1 Department of Biomedical Engineering, Huazhong University of Science and Technology, Wuhan, Hubei, China; 2 Raycan Technology Co., Ltd., Suzhou, Jiangsu, China; 3 Key Laboratory of Thermo-Fluid Science and Engineering, Ministry of Education, Xi’an Jiaotong University, Xi’an, China; Ural Federal University named after the first President of Russia B N Yeltsin Institute of Physics and Technology: Ural’skij federal’nyj universitet imeni pervogo Prezidenta Rossii B N El’cina Fiziko-tehnologiceskij institut, RUSSIAN FEDERATION

## Abstract

The energy information of pulse signals is significantly important for applications such as computed tomography (CT), positron emission tomography (PET), and research on defects in condensed matter. Time-over-threshold (TOT) and multi-voltage threshold (MVT) are commonly used digitization methods in sampling pulse signal. However, both approaches rely on a mathematical model of the pulse signal to derive energy information. This study proposes a semi-empirical mathematical model for pulse signals formation process in scintillation crystal-coupled photomultiplier tube(PMT) probes, by utilizing the CR-RC shaping method. This mathematical model accurately describes output of the PMT pulse signals. This study analyzes a substantial dataset of pulse signals, comparing the performance of the newly designed mathematical model with that of the double exponential function in terms of their ability to fit pulse signals. The results indicate that the mathematical model developed herein achieves an average *R*^2^ of 0.9255, significantly surpassing the 0.9155 of the double exponential function, thereby demonstrating its superior fitting efficacy.

## Introduction

Commonly used scintillation crystals are employed to detect *γ*-ray radiation and deposit the energy of *γ*-rays. When radiation interacts with a scintillator [1–3], it emits a multitude of photons proportionate to the radiation’s energy. Subsequently, these photons are converted into electrical pulse signals by a photodetector for processing by electronic devices. Pulse signals convey information such as the arrival times of high-energy particles, energy measurements, and scintillation crystal decay characteristics, among others. These signals find widespread applications in fields such as nuclear medicine, geological exploration, and high-energy physics.

The energy information of pulse signals is significantly important for applications such as computed tomography (CT), positron emission tomography (PET), and research on defects in condensed matter [4, 5]. The utilization of high-speed analog-to-digital converters (ADCs) enables precise energy quantification of pulse signals [6, 7]. However, this methodology is impractical in scenarios with an excessive quantity of channels due to elevated power consumption and costs. To circumvent these limitations, sampling method for the time at which a pulse signal crosses a voltage threshold has been developed, such as the time-over-threshold (ToT) method [8, 9] and the multi-voltage threshold (MVT) method [10–12]. The ToT approach estimates the signal’s energy by measuring the time difference as the pulse signal exceeds a certain voltage threshold, necessitating the utilization of information regarding the signal’s shape [5, 13, 14]. The MVT technique employs multiple voltage thresholds to sample the pulse signal, reconstructing the signal with a pre-determined pulse signal model to derive its timing and energy information [10, 15]. It utilizes the mathematical model of the signal to fit the data in order to obtain the signal waveform [10, 16, 17]. Thus, an accurate mathematical model for depicting the pulse signal is paramount.

The ToT and dynamic ToT methods utilize exponential functions as the mathematical models for pulse signals [14, 18]. The Dual Threshold ToT approach employs a more complex double exponential model [13]. The MVT frequently uses function models such as the linear-exponential composite function and the double exponential function [10, 19, 20]. These methodologies analyze the primary characteristics of pulse signals using approximate mathematical models. However, due to the imprecision of these mathematical models, the most accurate energy information cannot be achieved [15, 18, 21]. FlexToT converts the pulse shape into an approximate triangular wave, achieving improved linearity but losing other information like the pulse shape decay constant [22]. Hamby et al. designed a mathematical model for NaI(Tl) detection systems involving the convolution of multiple functions [20]. While this model can describe the pulse more accurately, it necessitates over ten parameters, implying the need for a substantial number of sampling points and thus placing high demands on the pulse signal acquisition circuit.

To address the above-mentioned issues, this study introduces a novel mathematical model for pulse signals by analyzing the signal characteristics in scintillator crystals coupled with photomultiplier tubes (PMTs). This study models the number of visible light photons within the crystal over time as a exponential signal. The transmission of a significant number of visible light photons through the optical coupler, where they are converted into electrical signals by the PMT, is akin to a signal passing through a low-pass filter. Drawing from this observation, the paper proposes a semi-empirical mathematical model for pulse signals, grounded in a exponential function, which undergoes multiple stages of low-pass filtering. This paper offers several significant contributions to the field: 1. It proposes a semi-empirical mathematical model that requires fewer parameters and is easier to use compared to commonly employed models. 2. The model can be utilized not only for the original pulse signals output by the PMT but also for the pulse signals after RC low-pass filter. 3. The model function provided in this paper offers a more precise description of the local details of the pulse signals.

## Methods

### Pulse signal transport in scintillation crystals, PMTs, and low-pass filters

Scintillation crystals are materials capable of absorbing high-energy radiation [23, 24]. When high-energy radiation interacts with crystals, the energy of high-energy rays is transferred to scintillation crystals through Compton effect, photoelectric effect, electron pair effect, etc. Sparkling crystals release energy in the form of a large number of visible photons. Scintillation crystals are characterized by their high density, rapid decay, high radiation resistance, high luminous efficiency, and superior optical properties. Presently, cerium-doped lutetium silicate crystals are esteemed in advanced PET imaging technologies for their high density, rapid decay, high luminous efficiency, and excellent radiation resistance [25–28]. Furthermore, LaBr_3_ is recognized for its exceptional light output, rapid decay time, and stable thermal properties, rendering it particularly noteworthy in various extreme environments [29, 30].

PMT serves as a sophisticated device designed to transform photons from visible light into measurable electrical signals [31]. In this process, photons first strike the cathode of the PMT, where they are converted into photoelectrons. These photoelectrons subsequently undergo amplification across multiple dynode stages, each stage contributing to a multiplication of secondary electrons. These secondary electrons are then accumulated at the anode, culminating in the production of an electrical output [32]. Characterized by its outstanding linearity, high gain, and exceptional sensitivity, the PMT is extensively employed across various scientific disciplines, including medicine, geology, and astronomy [31].

Low-pass filtering is a commonly employed method to eliminate high-frequency noise from signals. In circuits designed for processing electrical signals, a resistor and a capacitor can be easily utilized to construct such a filter. The cutoff frequency depends on the values of the resistor (R) and the capacitor (C). Additionally, systems processing non-electrical signals also exhibit low-pass filtering characteristics. A broader perspective is that, in the physical world, the vast majority of systems inherently possess some level of low-pass filtering characteristics [33].

When a gamma photon interacts with a scintillation crystal, it causes the scintillator to emit a large number of visible light photons. The variation in the quantity of these visible light photons over time is characterized by an exponential curve [34]. As a multitude of visible light photons emanates from the scintillation crystal and passes through an optical coupler to the PMT, this process—considering the low-pass filter characteristics inherent in physical systems—can be approximated as a low-pass filtering of the signal that represents the change in the quantity of visible light photons over time. A notable characteristic of the PMT’s photocathode is its signal broadening effect, which serves as a form of low-pass filtering. When visible light photons are converted into photoelectrons that are subsequently amplified to generate an electrical pulse signal, this pulse signal—characterized by its higher frequency components—undergoes a degree of low-pass filtering through the circuit elements it traverses. Additionally, resistors and capacitors may be used to design low-pass filters. Consequently, this study meticulously examines the comprehensive process encompassing the generation and collection of signals by the PMT.

### A semi-empirical mathematical model of pulse signal

Traditional mathematical models, including the exponential and bi-exponential models [20, 35], emphasize the primary characteristics of pulse signals while neglecting their localized variations. Furthermore, these models are typically applicable only to signals directly outputted by photomultiplier tubes (PMTs). When signals undergo further processing, such as filtering to enhance the signal-to-noise ratio, these models become unsuitable. This paper examines the complete signal flow, from the generation by scintillation crystals to the processing via circuit filters. Below, a semi-empirical mathematical model is proposed.

In this paper, the scintillation crystal, PMT, and circuitry are identified as components of a signal system, each functioning as a low-pass filter. We consider the curve representing the change in the number of visible light photons over time to be an exponential signal, as shown in (1) [34]:

$$\begin{array}{c} y\left(t\right)=Ae^{-\frac{t}{\tau }}\mu \left(t\right) \end{array}$$ (1)

Where *A* represents the peak value, and *τ* is the decay constant. Where *μ*(*t*) is the step function.

The response equation of the low-pass filter is illustrated as follows (2):

$$\begin{array}{c} h\left(t\right)=\frac{1}{\tau }e^{-\frac{t}{\tau }}\mu \left(t\right) \end{array}$$ (2)

Where *τ* denotes the time constant of the low-pass filter. In this context, we assume that the time constants of all filters align with the decay constant of the scintillation crystal.

Therefore, when filtering through a multi-stage low-pass filter, the waveform function model of the output is obtained by using integral transformation on Eqs (1) and (2). Reorganization of these parameters results in the expression shown in Eq (3) [21]:

$$\begin{array}{c} v\left(t\right)=at^{b}e^{ct}\mu \left(t\right) \end{array}$$ (3)

Where *a*, *b*, and *c* represent the parameters to be determined. In the model, parameter *a* is related to the signal amplitude and the order of the low-pass filter, parameter *b* is associated with the order of the low-pass filter, and parameter *c* pertains to the decay time of the pulse signal and the low-pass filter. This presentation clearly categorizes each parameter by its specific relationship and functionality, aligning with the precise and formal style typical in scientific publications.

The CR-RC method is one of the commonly used filtering techniques for pulse signals. It utilizes multi-stage RC low-pass filters to eliminate high-frequency noise from pulse signals. Consequently, the CR-RC circuit can effectively enhance the signal-to-noise ratio. This improvement plays a crucial role in enhancing the energy resolution performance of detectors. Furthermore, it can shape the pulse signals, modifying their characteristics to facilitate subsequent processing and analysis.

Referring to the equation of the CR-RC shaped signal, the parameter *b* in Eq (3) is traditionally considered an integer corresponding to the order of filtering. However, given that the temporal profile of visible light photon counts does not strictly follow an exponential function, the time constants associated with various segments of the signal filtering differ. Consequently, considering the parameter *b* as a mere integer may not represent the optimal approach. Employing a semi-empirical approach, we have expanded the range of *b* to include real numbers.

## Experimental setup

**Fig 1:**
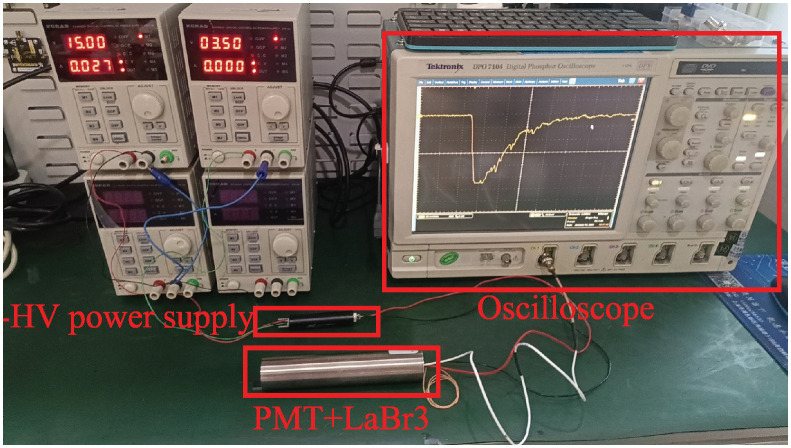
Signal acquisition platform.

**Fig 2:**
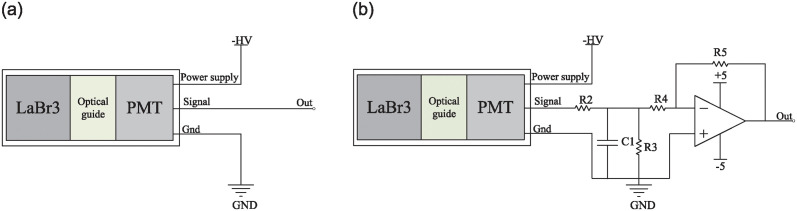
Signal Readout Diagram: (a) Signal directly connected to the oscilloscope; (b) Signal after filtering and reverse amplification connected to the oscilloscope.

As illustrated in Fig 1, the probe employed to acquire scintillation pulse signals comprises a lanthanum bromide scintillation crystal coupled with a PMT(R1288A, Hamamatsu). A high-speed oscilloscope(DPO7104, Tektronix) is employed for acquiring scintillation pulse signal waveforms for subsequent analysis. As illustrated in Fig 2, both the raw pulse signals and the waveforms post-RC low-pass filtering are captured. The collection of raw scintillation pulse signals involves directly inputting signals from the PMT output to the high-speed oscilloscope via a coaxial line with a characteristic impedance of 50 Ω. Conversely, the acquisition of RC filtered scintillation pulse signals involves feeding PMT output signals into an RC low-pass filter via a coaxial line with a characteristic impedance of 50 Ω, followed by reverse amplification (AD8000, ADI). Subsequently, the amplified signals are fed into the high-speed oscilloscope through a coaxial line with a characteristic impedance of 50 Ω. In both methods, the oscilloscope is configured with a 50 Ω load, a 10 GS/s sampling rate, and a 1 GHz analog bandwidth. This setup ensures precise measurement and analysis of scintillation pulse signals, essential for accurate experimental results. The use of the high-speed oscilloscope facilitates the detailed observation of signal characteristics and temporal resolution, crucial for understanding the dynamics of the scintillation process.

**Fig 3:**
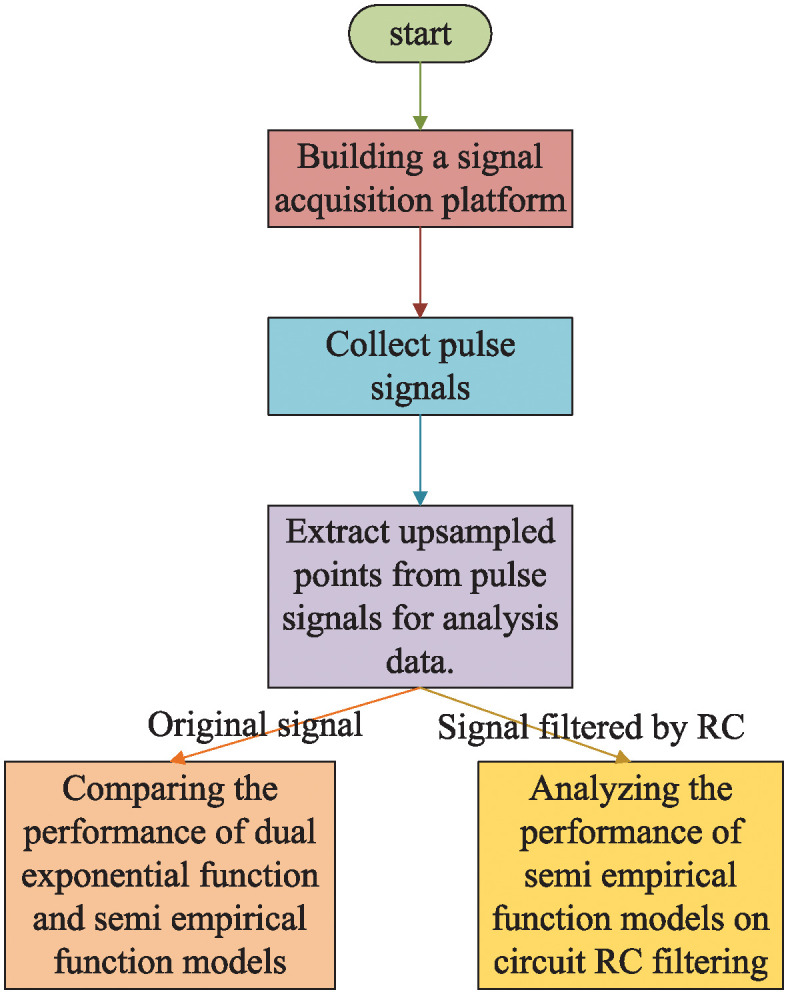
Experimental flowchart.

The experimental protocol of this study is depicted in Fig 3. Data points were extracted from pulse signals captured by a high-speed oscilloscope by setting a voltage threshold, facilitating subsequent analysis. This study collected both the raw pulse signals directly emitted from the PMT and the signals after RC filtering. The initial type of pulse signal facilitated the analysis and comparison of the bi-exponential and Eq (3) functions’ performances. The second type of pulse signal was employed to assess the Eq (3) function’s efficacy on the filtered signals.

## Results

Negative pulse signals are inverted into positive signals to facilitate analysis. In this experiment, a voltage threshold of 15 mV is established, and only data points from scintillation pulses exceeding this threshold are utilized as validation data for the fitting function. Signals directly output by the PMT are fitted with both Eq (3) and a bi-exponential function, yielding fitted pulse signals and *R*^2^ outcomes. Bi-exponential function are as defined in Eq (4):

$$\begin{array}{c} v\left(t\right)=\left(me^{nt}+pe^{qt}\right)\mu \left(t\right) \end{array}$$ (4)

The parameters *m*, *n*, *p*, and *q* are to be determined. Furthermore, since pulse signals post-filtering are unsuitable for bi-exponential function fitting, signals output through low-pass filters are exclusively fitted using Eq (3), yielding fitted pulse signals, *R*^2^ results, and equation parameters.

Meanwhile, we also employed a linear-exponential function to fit the raw pulse signals [10]. The linear-exponential function is one of the commonly used model functions for scintillation pulse signals and has been widely applied in PET systems [10]. The linear-exponential function is expressed as shown in Eq (5):

$$\begin{array}{c} v\left(t\right)=\{\begin{array}{cc} 0 & t<0,\\ V_{p}\left(t/t_{p}\right) & 0<t<t_{p},\\ V_{p}e^{-}(t-t_{p})/\tau & t\geq t_{p}, \end{array} \end{array}$$ (5)

Where *V*_*p*_ represents the peak value of the pulse signal, and *t*_*p*_ denotes the time at which the peak occurs. Since the linear-exponential function fits different segments of the signal with a linear function and an exponential function, the final *R*^2^ value is obtained by calculating a weighted average of the two individual *R*^2^ values based on the number of sampling points in each segment. For example, if the sampling points of the linear portion constitute 10% of the total points with an *R*^2^ value of $R_{1}^{2}$, and the exponential portion accounts for 90% with an $R_{2}^{2}$ value, the overall *R*^2^ value is calculated as $R^{2}=0.1R_{1}^{2}+0.9R_{2}^{2}$.

**Fig 4:**
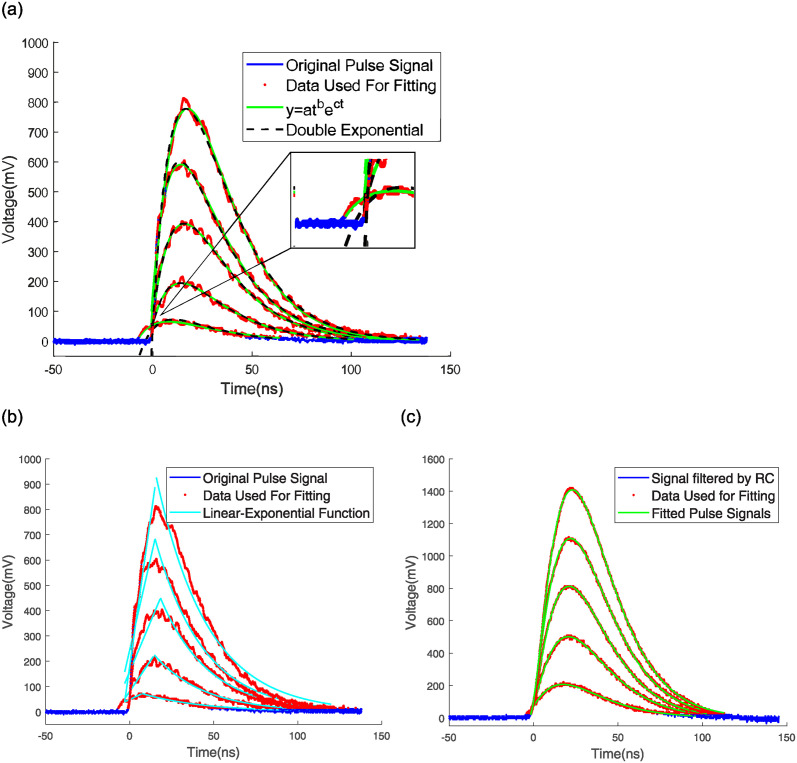
The original pulse signal is used to reconstruct the pulse signal waveform using Eq (3) and the double exponential function(a), and to reconstruct the pulse signal waveform using the linear exponential function(b). Reconstruct the waveform of the filtered pulse signal using Eq (3)(c).

As shown in Fig 4, we analyzed multiple pulse signals fitting outcomes derived from an extensive dataset comprising both directly emitted and processed pulse signals. In the (a) and (b) panel, the blue lines depict the waveforms of the original scintillation pulses emitted by the PMT, with amplitudes of 72 mV, 216 mV, 404 mV, 604 mV, and 812 mV. Conversely, in the (c) panel, the blue lines represent the waveforms of the pulses signals post-processing through an RC low-pass filter and inversion, with amplitudes of 220 mV, 508 mV, 812 mV, 1116 mV, and 1420 mV. The red dots in both panels denote the data points utilized for validation of the fitting algorithm. The green lines indicate the pulse signals modeled by fitting the red data points using Eq (3), while the yellow lines represent the pulse signals derived from fitting these points via a bi-exponential function. And the cyan blue line represents the pulse signal obtained by fitting these points with linear-exponential function. The analysis demonstrates that the fitting outcomes from Eq (3) more closely approximate the original pulse signal, particularly in the initial phase. The significant overlap between the original pulses captured by the oscilloscope and the modeled pulse signals underscores the accuracy of our fitting functions in describing the pulse signals.

**Fig 5:**
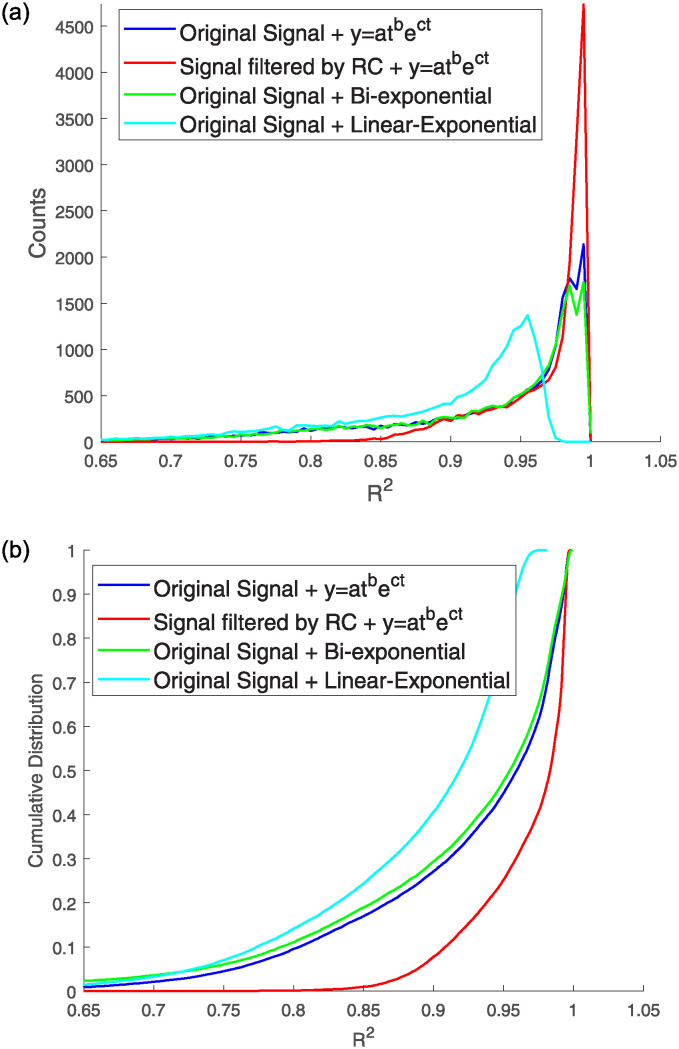
Histogram of *R*^2^ values for model fits to pulse data (a). Cumulative distribution of *R*^2^ values for model fits to pulse data (b).

In this study, we analyze the fitting results of 20,000 pulse signals, both original output signals and filtered signals by RC low-pass filter, to obtain the *R*^2^ values. Fig 5 presents a histogram illustrating the distribution of count values of *R*^2^ and a cumulative distribution plot of *R*^2^. Table 1 presents the mean values of *R*^2^, illustrating that the distribution of these values, when fitted with three distinct functions, demonstrates that the results obtained with Eq (3) are more concentrated and closer to 1. This concentration suggests a more accurate description of the pulse signals. When fitted with Eq (3), the *R*^2^ values for both the original and filtered signals cluster around 1, with the mean, median, and mode all closely approaching 1. This clustering suggests that Eq (3), a composite function, can accurately describe scintillation pulses. In Fig 5, the *R*^2^ values for the filtered signals exceed 0.8 and are more concentrated around 1, whereas the original output signals’ *R*^2^ values range between 0.65 and 1, indicating a more dispersed distribution. Combining Figs 4 and 5, it can be seen that the linear exponential function has a slightly insufficient degree of fitting to the pulse signal compared to the other two functions.

**Table 1:** Mean, median, and mode of *R*^2^.

*R* ^2^	Mean	Median	Mode
Original Signal + Linear-Exponential	0.8889	0.9185	0.9518
Original Signal + *y* = *at*^*b*^*e*^*ct*^	0.9255	0.9594	0.9939
Signal filtered by RC + *y* = *at*^*b*^*e*^*ct*^	0.9643	0.9821	0.9993
Original Signal + Bi-exponential	0.9155	0.9544	0.9957

Moreover, the mean, median, and mode of the filtered signals are all higher than those of the original output signals. This improvement is attributed to the filtering process, which removes most of the noise from the scintillation pulses, resulting in a higher signal-to-noise ratio and a smoother signal shape. Consequently, this function model can precisely describe both the original and filtered signals, as filtering effectively removes noise, allowing the waveforms to more closely match the fitting function curves. The cumulative distribution plot for *R*^2^ presented in Fig 5 clearly demonstrates that the bi-exponential fits exhibit the highest cumulative distributions, indicative of poorer fitting results. Pulse signals with intermediate cumulative distributions, as seen in the original measurements, demonstrate better fitting accuracy. Conversely, pulse signals that have been filtered through an RC low-pass filter exhibit the lowest cumulative distributions, indicative of the most accurate fitting results.

**Fig 6:**
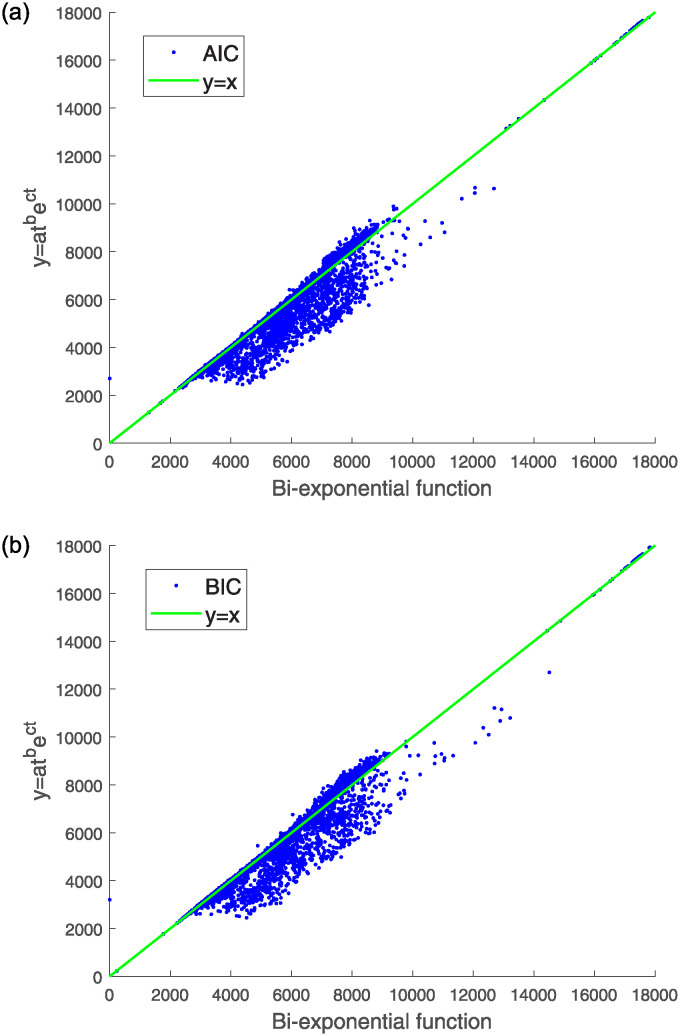
AIC (a) and BIC (b) values obtained from fitting and reconstruction of the pulse signal using the *y* = *at*^*b*^*e*^*ct*^ and bi-exponential function.

Compared to the linear exponential function, the double exponential function and Eq (3) are more accurate in fitting pulse signals. Therefore, we will evaluate the double exponential function and the results of Eq (3) based on indicators such as AIC (Akaike Information Criterion) and BIC (Bayesian Information Criterion). When reconstructing the original signal using the bi-exponential function and Eq (3), this paper also calculated the AIC and BIC values for the two models fitted to the same pulse signal. AIC and BIC are commonly used criteria in statistical model selection, mainly to evaluate the goodness of fit and choose the optimal model. AIC takes into account both the goodness of fit and the complexity of the model, with a smaller AIC value indicating better model performance. BIC is similar to AIC but imposes a greater penalty for model complexity. Like AIC, a lower BIC value indicates a better model. As shown in Fig 6, a scatter plot was generated with the AIC values of the double exponential function on the x-axis and the AIC values of Eq (3) on the y-axis. A similar scatter plot was created for the BIC values. The results show that most of the scatter points are concentrated below the *y* = *x* line, indicating that both the AIC and BIC values of Eq (3) are lower than those of the double exponential function.

**Fig 7:**
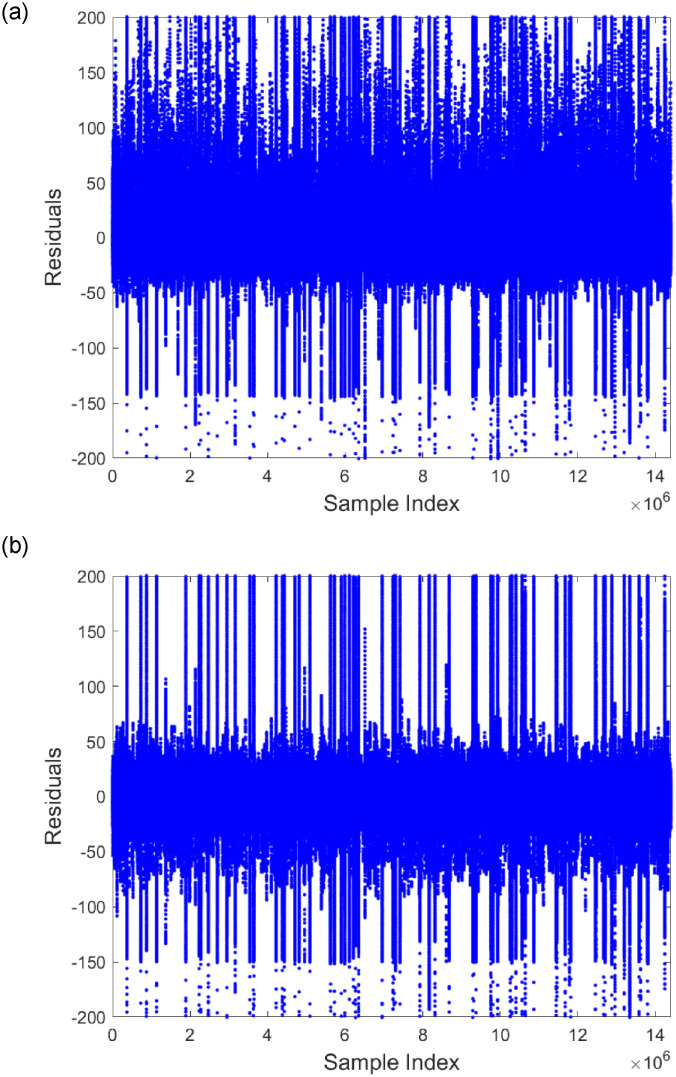
Scatter plot of residuals versus sampling points for all pulse signals, with residuals obtained from reconstruction using the bi-exponential function (a) and the *y* = *at*^*b*^*e*^*ct*^ function (b).

In addition, the paper calculated the residuals for the signal reconstruction using both the double exponential function and Eq (3). As shown in Fig 7, all pulse signal sampling points are ordered by index, with the x-axis representing the index and the y-axis representing the residual value of each sampling point. It can be observed that the residuals computed using the double exponential function are concentrated in the range of [−50, 100], whereas those computed using Eq (3) are concentrated in the range of [−50, 50].

**Fig 8:**
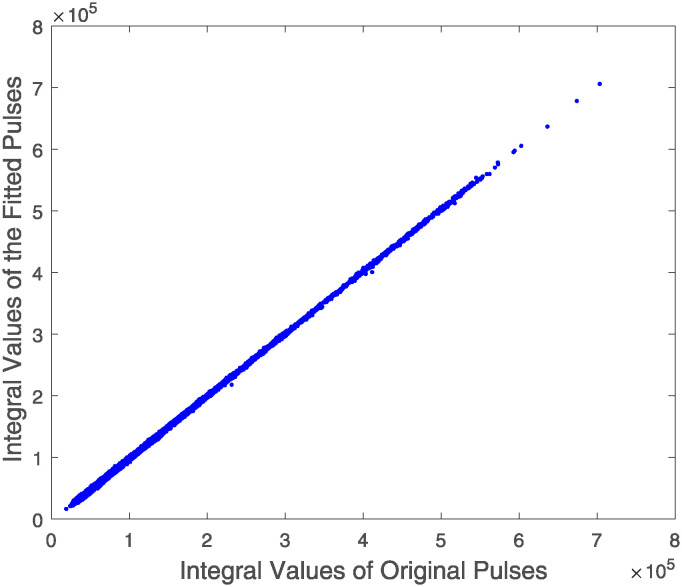
Scatter plot of integrated values of original signals versus fitted pulse integrals.

Additionally, we correlate the integral values of the filtered pulse signals with those reconstructed using Eq (3), illustrating this relationship through a scatter diagram as depicted in Fig 8. Within the energy range of the sampled data, the linearity of the energy is consistently maintained.

**Fig 9:**
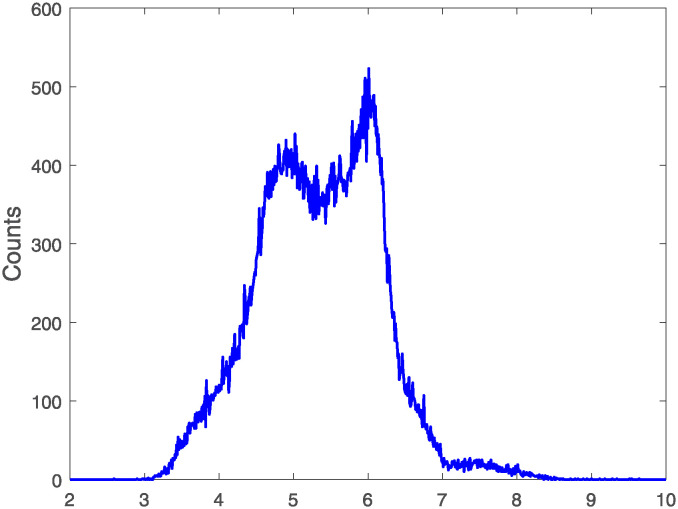
Histogram of parameter *b*.

As illustrated in Fig 9, parameters *b*, derived from the mathematical model applied to the filtered scintillation pulses, are extracted and represented in a histogram. It is apparent from the figure that the distribution of parameter *b* encompasses a specific range, rather than comprising discrete integers. This result indicates that expanding the range of values for parameter *b* proves effective.

## Conclusion and discussion

In this study, we introduce a mathematical model for the pulse signal output of PMTs, which is designed based on the characteristics of low-pass filtering. This model not only demonstrates enhanced accuracy compared to the double exponential model but also shows superior adaptation to the shape of pulse signals filtered through additional low-pass filters. Furthermore, the model exhibits a concise configuration with fewer parameters, potentially reducing the required computational resources. It is readily applicable in diverse scenarios that require streamlined computations and demonstrates extensive applicability in various energy spectrum usage contexts. Eq (3) takes the form of a gamma function distribution, where parameter *b* affects the shape of the signal waveform, particularly the position of the peak and the sharpness of the waveform. Parameter *c* influences the width of the signal waveform. Expanding the range of *b* from positive integers to rational numbers enhances the flexibility of this function for pulse signal reconstruction.

However, the model presented herein lacks a rigorous mathematical derivation and has been exclusively validated through experiments, indicating a closer alignment with empirical pulse models. Additionally, its application has been confined to PMT signals and it remains untested with other types of photodetectors. Therefore, the functional model discussed in this paper has the potential to significantly contribute to future applications involving PMTs, including monitoring in proton therapy, geological exploration, elemental analysis, and high-energy radiation detection in nuclear physics. Moreovre, the semi-empirical mathematical model proposed in this paper demonstrates strong performance with only three parameters, rendering it suitable for acquisition systems with a limited number of voltage thresholds, such as two. The model’s simplicity allows for transforming the function into an additive form by taking logarithms, thereby circumventing the need for iterative fitting using nonlinear least squares, a process that demands extensive computational resources. Alternatively, the model utilizes linear least squares, which impose lower computational demands. This characteristic potentially facilitates the implementation of the function in FPGA for real-time computation. Moreover, the function model can be simplified to an exponential form, presenting a novel approach that could advance research in scintillation detectors. Therefore, the detection research of the function model obtained in this article in pulse signals will be the focus of our future work.
